# DYRK1A phosphorylates MEF2D and decreases its transcriptional activity

**DOI:** 10.1111/jcmm.16505

**Published:** 2021-06-09

**Authors:** Pin Wang, Juan Zhao, Xiulian Sun

**Affiliations:** ^1^ NHC Key Laboratory of Otorhinolaryngology Qilu Hospital Cheeloo College of Medicine Shandong University Jinan China; ^2^ Department of Otorhinolaryngology Qilu Hospital of Shandong University Jinan China; ^3^ Brain Research Institute Qilu Hospital of Shandong University Jinan China; ^4^ Key Laboratory of Cardiovascular Remodeling and Function Research Chinese Ministry of Education Chinese National Health Commission Qilu Hospital of Shandong University Jinan China

**Keywords:** DYRK1A, glioblastoma, MEF2D, phosphorylation, transcriptional activity

## Abstract

Myocyte enhancer factor 2D (MEF2D) is predominantly expressed in the nucleus and associated with cell growth, differentiation, survival and apoptosis. Previous studies verified that phosphorylation at different amino acids determined MEF2's transcriptional activity which was essential in regulating downstream target genes expression. What regulates phosphorylation of MEF2D and affects its function has not been fully elucidated. Here, we uncovered that dual‐specificity tyrosine phosphorylation regulated kinase 1A (DYRK1A), a kinase critical in Down's syndrome pathogenesis, directly bound to and phosphorylated MEF2D at Ser251 in vitro. Phosphorylation of MEF2D by DYRK1A significantly increased MEF2D protein level but attenuated its transcriptional activity, which resulted in decreased transcriptions of MEF2D target genes. Phosphorylation mutated Ser251A MEF2D exhibited enhanced transcriptional activity compared with wild type MEF2D. MEF2D and DYRK1A were observed co‐localized in HEK293 and U87MG cells. Moreover, DYRK1A‐mediated MEF2D phosphorylation in vitro might influence its nuclear export upon subcellular fractionation, which partially explained the reduction of MEF2D transcriptional activity by DYRK1A. Our results indicated that DYRK1A might be a regulator of MEF2D transcriptional activity and indirectly get involved in regulation of MEF2D target genes.

## 1 INTRODUCTION

MEF2D is a member of MEF2 protein family, a transcription factor family containing MEF2A, MEF2B, MEF2C and MEF2D. MEF2 plays critical roles in cell development.^1^, ^2^ They participate in many signal pathways relevant to cell division, apoptosis and differentiation, involving MAPK1,^3^ CDK5,^4^ MAPK7,^5^ and AMPK.^6^ Many studies manifest MEF2D participating in tumorigenesis and cancer progression. MEF2A and MEF2D are up‐regulated during differentiation of mouse embryonal carcinoma P19 cells, while MEF2B is highly expressed in undifferentiated P19 cells.^7^ Elevated MEF2D expression is detected in hepatocellular carcinoma clinical specimens, especially in those with poor prognosis.^8^ MEF2D is also observed abundant in lung cancer tissues and cell lines comparing with the matched normal tissues and cell lines. Knocking down of MEF2D by miRNA interference suppresses the growth of lung carcinoma.^9^ Silencing of MEF2D triggers G2‐M arrest in a way associated with direct down‐regulation of genes related to cell cycle progress in hepatocellular carcinoma.^8^, ^10^ Inflammatory conditions increase MEF2D expression in lung cancer cells, which might contribute to cancer development by influencing cancer microenvironment and cell bio‐behaviours.^11^ Increased MEF2D expression exists in pancreatic cancer tissues compared with adjacent normal tissues. MEF2D regulates cell proliferation, migration and invasion abilities in pancreatic cancer via Akt/GSK‐3β signalling pathway. Of note, the increased expression of MEF2D is associated with tumour size, histological differentiation and TNM stage among pancreatic cancer patients.^12^ In colorectal cancer tissues, MEF2D is positively correlated with CD31‐positive microvascular density and promote tumour angiogenesis in vitro and in vivo, resulting in induction of expression of proangiogenic cytokines.^13^ Our previous studies showed that DYRK1A is up‐regulated by MEF2D, leading to decreased expression of NFATc2, a substrate of DYRK1A,^14^ and a regulator of glioblastoma invasion.^15^ MEF2D tumour‐associated activity could be suppressed by miR‐421 in gliomas.^16^ Proliferations and invasion of cervical cancer cells might be regulated by miR‐30a which also targets MEF2D.^17^ Collectively, expression of MEF2D is vitally important to cancer pathogenesis and progression.

Transcriptional activity of MEF2D could be partially modulated by post‐translational modification. Phosphorylation of MEF2D was intensively investigated. Intriguingly, phosphorylation at different amino acid residues has diverse effects on MEF2D transcriptional activity. For instance, phospho‐Ser179 induced by MAPK7 increased transactivation of MEF2D in Hela cells.^18^ ATM phosphorylates and activates MEF2D, contributes to neuronal survival in response to DNA damage.^19^ In contrast, MEF2A and MEF2D were phosphorylated with the induction of apoptosis of cerebellar granule neurons. But sites phosphorylated during apoptosis are functionally distinct. The increased phosphorylation of MEF2A and MEF2D leads to decreased DNA binding ability and reduced transcriptional activity.^20^ Removal of depolarization induces hyperphosphorylation of MEF2D at certain serine/threonine residues, following with weakened DNA binding ability and increased susceptibility to caspases.^21^ Phospho‐Ser444 by Cdk5 inhibits MEF2D transcriptional activity, while mutated MEF2D resistant to Cdk5 phosphorylation restores transcriptional activity and protects primary neurons from apoptosis induced by Cdk5 and neurotoxin.^4^, ^22^ Phosphorylation by PKA and GSK3β represses transactivation properties of MEF2D as well.^23^, ^24^ As a transcription factor, MEF2D is predominantly expressed in the nucleus. Early study shows modulation of MEF2D activity by chaperon‐mediated autophagy. By interacting with Hsc70, MEF2D is observed shuttling from the nucleus to the cytoplasm to undergo degradation in mouse midbrain dopaminergic progenitor cells.^25^ MEF2D subcellular localization might also change upon cell stress. Rotenone, a neurotoxin, induced an up‐regulation of MEF2D in the nucleus which might be protective against mitochondrial dysfunction and oxidative stress.^26^ MEF2D level in both the cytoplasm and the nucleus is lower in nigral neurons of PD patients than that of AD and the control group, probably associating with α‐synuclein aggregates.^27^ Given modulation of MEF2 activity is partially determined by phosphorylation at distinct sites, phosphorylation of MEF2D contributing to its export from the nucleus deserves consideration.

Dual‐specificity tyrosine‐regulated kinase 1A (DYRK1A), abundant in neuronal cells, is widely involved in a variety of diseases including human malignancies, such as haematological and brain cancers.^28^, ^29^, ^30^, ^31^ DYRK1A was observed to accumulate in a punctate pattern in the nucleus of transfected cos‐7 and HEK293 cells.^32^ Identified as regulators of NFAT, DYRK1 and DYRK2 regulate NFAT subcellular localization by directly phosphorylating the conserved serine‐proline repeats3 (SP‐3) motif of the NFAT regulatory domain, exerting phosphorylation of SP‐2 and serine‐rich region 1(SRR‐1) motifs by GSK3 and CK1.^33^ DYRK1A phosphorylates GLI1 at Ser408 and may facilitate GLI1 nuclear localization.^34^, ^35^ Cytoplasmic CDKL5 expression is decreased upon phosphorylation at Ser308 by DYRK1A.^36^ DYRK1A phosphorylates FKHR at Ser329 and reduces the proportion of FKHR in the nucleus.^37^ Obviously, phosphorylation state of protein strongly affects its subcellular localization. Our previous study showed that DYRK1A was degraded by E3 ligase SCF^βTRCP^.^38^ We also proved that DYRK1A phosphorylates NFATc1 and increases its protein stability.^39^ Recently, we showed that DYRK1A phosphorylates and increases insulin receptor substrate‐1 expression and regulates insulin signalling.^40^ We previously demonstrated that MEF2D up‐regulated expression of DYRK1A through binding to its responsive element in DYRK1A promoter region and enhancing its transcriptional activity.^14^ Their expressions were strongly correlated during mice brain development. The kinase activity of DYRK1A may also be induced by MEF2D in glioblastoma cell lines.^14^ Therefore, interaction of DYRK1A and MEF2D needs to be further addressed. Here we demonstrate that DYRK1A significantly increases MEF2D protein expression. DYRK1A and MEF2D are co‐localized mainly in the nucleus. DYRK1A directly binds to and phosphorylates MEF2D at Serine 251, reducing its transcriptional activity. DYRK1A affects MEF2D subcellular localization without affecting its protein stability. These results confirm the interaction between DYRK1A and MEF2D, and highlight their potential roles in glioblastoma development.

## 2 MATERIALS AND METHODS

### 2.1 Cell culture and transfection

HEK293 cells and U87MG cells were cultured in Dulbecco's modified Eagle's medium containing 10% fetal bovine serum, 4.5 g/L glucose, 1 mmol/L sodium pyruvate, 2 mmol/L l‐glutamine, 25 mmol/L HEPES, 100 units/mL penicillin and 0.1 mg/mL streptomycin. Cells were maintained at 37°C in an incubator containing 5% CO_2_. Harmine (Aladdin) was added into culture medium of U87MG cells for 24 hours with concentration at 10 μmol/L.

For transfection, cells were seeded into cell culture dishes or plates with cell confluency of 50%. When cell confluency reached approximately 80% next day, plasmids were transfected into cells by VigeneFection transfection reagent (Vigene Biosciences) and lipofectamine™ 2000 Transfection Reagent (Thermofisher) according to manufacturer's instructions.

### 2.2 Western blotting and antibodies

Cells were harvested in 48 hours of transfection and washed with ice‐cold PBS twice. 0.1% SDS‐RIPA lysis buffer (Beyotime Institute of Biotechnology, Haimen, China) was used as cell lysis buffer in the presence of protease inhibitor cocktail mixture. Protein concentration was measured by DC protein assay kit (Bio‐Rad) and Pierce™ BCA Protein Assay Kit. Protein samples were separated by 10% and 12% glycine SDS‐PAGE. PageRuler pre‐stained protein ladder (Thermofisher, Waltham, USA) was used to indicate protein molecular weights. Proteins were transferred onto nitrocellulose membrane under 60 V for 3 hours. Nitrocellulose membranes were then blocked in 5% BSA in TBS‐T for 4 hours at room temperature and then incubated in primary antibody dilutions overnight at 4°C. After washing three times in TBS‐T for 5 minutes each, membranes were incubated in fluorescence‐conjugated secondary antibody dilutions for 20 minutes at room temperature. Signals were detected on LI‐COR Odyssey Infrared Imaging System (LI‐COR Biosciences, Lincoln, NE). Primary antibodies used in this study were as follows: FLAG antibody (Sigma); DYRK1A antibody (Sigma); GAPDH antibody (PTG); β‐actin antibody (Sigma); MEF2D antibody (BD). IRDye 680RD goat anti‐mouse IgG, IRDye 680RD goat anti‐rabbit IgG, IRDye 800CW goat anti‐mouse IgG and IRDye 800CW goat anti‐rabbit IgG were all purchased from LI‐COR Biosciences.

### 2.3 Co‐immunoprecipitation assay

Cells were harvested and lysed in Western and IP Cell lysis buffer which contains 20 mmol/L Tris (pH 7.5), 150 mmol/L NaCl, 1% Triton X‐100 in the presence of protease inhibitor mixture (Roche Applied Science). Cell lysate was centrifuged at 22000 *g* at 4°C for 15 minutes. Supernatant was carefully transferred into a new 1.5‐mL tube. Whole cell lysate containing 100 μg protein was used as input. Primary antibodies and protein A/G‐agarose beads (Santa Cruz Biotechnology, Santa Cruz, CA) were added into cell lysate and left on tube revolver at 4°C overnight. Mouse IgG (Beyotime Institute of Biotechnology, Haimen, China) was used as negative control. After incubation, agarose beads were pelleted by centrifuging at  800 *g* for 5 minutes at 4°C. Pellets were washed with western and IP cell lysis buffer once and ice‐cold PBS twice, respectively. Pellets were resuspended in loading buffer (Beyotime Institute of Biotechnology, Haimen, China) and denatured at 95°C for 5 minutes. Samples were analysed on 10% glycine SDS‐PAGE.

### 2.4 Plasmids and siRNA

pCMV6‐entry‐MEF2D (RC208748) and pCMV6‐entry‐DYRK1A (RC213183) expression plasmids were purchased from OriGene Technologies. pEnter‐DYRK1B expression plasmid (CH871514) was obtained from Vigene Biosciences. MEF2D and DYRK1A coding sequences were cloned into pDsRed‐Express1 and pEGFP‐N2 to generate MEF2D‐RFP and pEGFP‐DYRK1A. 3xMRE‐luc was kindly provided by Dr Michael E. Greenberg's lab. 3xMRE‐luc vector has three repeats of MEF2 response element (CTAAAAATAG) as previously described.^41^, ^42^ Primers designed for construction of pET28b‐MEF2D were as followed: reverse, 5′‐CGGAATTCGATGGGGAGGAAAAAGATT‐3′; reverse, 5′‐CCCAAGCTTCCACTTTAATGTCCAGGT‐3′. MEF2DS251A vector was generated by site‐directed mutation of MEF2D expression vector at Ser251 (S‐A). The following primers were used: MEF2DS251A, forward, 5′‐ATCCCTGCCAAGGCTCCACCCCCACCTACC‐3′; MEF2DS251A, reverse, 5′‐GGTAGGTGGGGGTGGAGCCTTGGCAGGGAT‐3′; MEF2DS251D, forward, 5′‐ATCCCTGCCAAGGATCCACCCCCACCTACC‐3′; MEF2DS251D, reverse, 5′‐GGTAGGTGGGGGTGGATCCTTGGCAGGGAT‐3′. DYRK1A‐KD vector was constructed by site mutation of DYRK1A expression vector at Lys188 (K‐R).^43^ Primers were as follows: DYRK1AK188R, forward, 5′‐ATGGGTTGCCATTAGAATAATAAAGAACAA‐3′; DYRK1AK188R, reverse, 5′‐TTGTTCTTTATTATTCTAATGGCAACCCAT‐3′. DYRK1A siRNA was purchased from GenePharma (Shanghai, China), and the sense sequences were as follows: siDYRK1A: 5′‐AAACUCGAAUUCAACCUUATT‐3′, negative control: 5′‐UUCUCCGAACGUGUCACGUTT‐3′. All plasmids were sequenced for validation.

### 2.5 In vitro kinase assay

Recombinant human DYRK1A protein was purchase from Thermo Fisher Scientific Inc Recombinant human MEF2D protein was purified from BL21(DE3) *Escherichia coli* as previously described.^44^ MEF2D (3 μg) was incubated with recombinant human DYRK1A protein (1 μg) in kinase buffer (25 mmol/L Tris‐HCl pH7.5 plus phosphatase inhibitors) with ATP (1 mmol/L) in metal bath at 30°C for 30 minutes. Reaction solution was analysed on polyacrylamide gel.

### 2.6 Site‐directed mutagenesis

Briefly, primers covering mutated sites were designed to amplify linear fragment. pCMV6‐entry‐MEF2D and pCMV6‐entry‐DYRK1A vectors were used as the templates for PCR. PCR products were digested with DpnI at 37°C for 1‐2 hour and transfected into DH5α competent cells. Single bacteria colony was amplified in LB media. Plasmid was purified and sent for sequencing.

### 2.7 Real‐time quantitative PCR

Total RNA was isolated from U87MG cells by TRIzol reagent (Sigma). 10‐40 cycles of PCR were performed to cover the linear range of the PCR amplification. The real‐time quantitative PCR was achieved by ABI 7900HT Fast Real‐time PCR system (Applied Biosystems) with SYBR^®^ Green‐based gene expression analysis. A comparative CT method (2^−ΔΔCT^) was used to analyse the gene expression level. The sequences of primers for real‐time quantitative PCR were listed in Table 1.

**TABLE 1:** Table of primers used for quantitative PCR

Genes		Sequence
DYRK1A	Forward	GGATCGTTACGAAATTGACTCCT
	Reverse	ACATAAAGTGGCGTTTCAAATGC
DYRK1B	Forward	CACCCCAGGATTCGAGCAAC
	Reverse	TGAGCGAGTCAATTTCGTAGC
HDAC9	Forward	AGTAGAGAGGCATCGCAGAGA
	Reverse	GGAGTGTCTTTCGTTGCTGAT
MEF2D	Forward	CGTGCTATGTGACTGCGAGAT
	Reverse	CAGCAGGGGGCTCTGTTCCAG
ZEB1	Forward	GCCAATAAGCAAACGATTCTG
	Reverse	TTTGGCTGGATCACTTTCAAG
β‐actin	Forward	GACAGGATGCAGAAGGAGATTACT
	Reverse	TGATCCACATCTGCTGGAAGGT

### 2.8 Immunofluorescence

HEK293 and U87MG cells were fixed and immunostained with mouse anti‐DYRK1A (Sigma), rabbit anti‐MEF2D (Proteintech). Goat Anti‐Mouse IgG(H + L), CoraLite488 conjugate (Proteintech) and goat Anti‐Rabbit IgG(H + L), CoraLite594 conjugate (Proteintech) antibodies were used to amplify and display the signals. DAPI (1 μg/mL; Roche Applied Science) was applied in mounting medium to indicate the nucleus. The images were captured by a fluorescence confocal microscope (LSM880, ZEISS). The analysis was achieved by ImageJ.

### 2.9 Luciferase assay

HEK293 cells and U87MG cells were seeded into 48‐well plates and transfection was performed next day. Cells were lysed 48 hours after transfection. Dual‐luciferase assays were achieved following the protocol supplied by dual‐luciferase reporter assay kit (Promega). Absorbance value was detected by GloMax^®^ 20/20 luminometer. Firefly luciferase activity was standardized by renilla luciferase activity and expressed as relative luciferase units.

### 2.10 Mass spectrometry analysis

Sample Mixture was separated on 10% glycine gel at 120 V for 90 minutes. Upon electrophoresis, proteins were fixed within a polyacrylamide matrix by incubating the entire gel in 5% (vol/vol) acetic acid in 1:1 (vol/vol) water:methanol. Coomassie Brilliant Blue (0.05% in 5% (vol/vol) acetic acid in 1:1 (vol/vol) water:methanol solution) was used for gel staining. After destaining, bands were cut out and in‐gel digestion was performed as described previously.^45^ Mass spectrometry analysis was performed at Shanghai Institutes for Biological Sciences. M/z values of cleaved peptides were examined and calculated. Mascot was used to identify phosphorylated peptide and their non‐phosphorylated counterparts.

### 2.11 Subcellular fractionation

Nuclear and cytoplasmic extraction kit was purchased from BestBio Science. Subcellular fractionation was performed according to manufacturer's instruction. Briefly, cells were collected and washed in cold PBS. Supernatant was removed and discarded. Cell pellet was resuspended in extraction solution A on ice for 15 minutes with vortex at 5 minutes intervals. After the final vortex for 5 seconds, cell solution was centrifuged at 16 000 *g* for 5 minutes at 4°C. Supernatant was transferred into a new Eppendorf tube as cytoplasmic fraction. The pellet was washed with cold PBS and resuspended in extraction solution B on ice for 40 minutes with vortex at 10 minutes intervals. Cell solution was centrifuged at 16 000 *g* for 10 minutes at 4°C. Supernatant was transferred into a new Eppendorf tube as nuclear fraction.

### 2.12 Cycloheximide (CHX) pulse‐chase assay

CHX pulse‐chase assay was performed as previously described.^38^ Briefly, HEK293 cells were transfected with MEF2D and MEF2D mutant vectors, respectively. Twelve hours after transfection, HEK293 cells were seeded in 6‐well plates. Thirty‐six hours after transfection, cells were treated with 150 μg/mL CHX and harvested every 12 hours for Western blotting analysis.

### 2.13 Statistical analysis

Data are presented as mean ± standard deviation (SD) from three independent experiments. For immunoblotting, one representative picture was shown. Quantifications from three independent experiments were defined with blots density by ImageJ software. The data were evaluated analysed for statistical significance with analysis of variance or non‐parametric analysis by Prism 7 (GraphPad Software, Inc, San Diego, CA, USA). Differences were classified as significant at *P* < .05.

## 3 RESULTS

### 3.1 DYRK1A phosphorylates MEF2D and increases MEF2D expression

**FIGURE 1:**
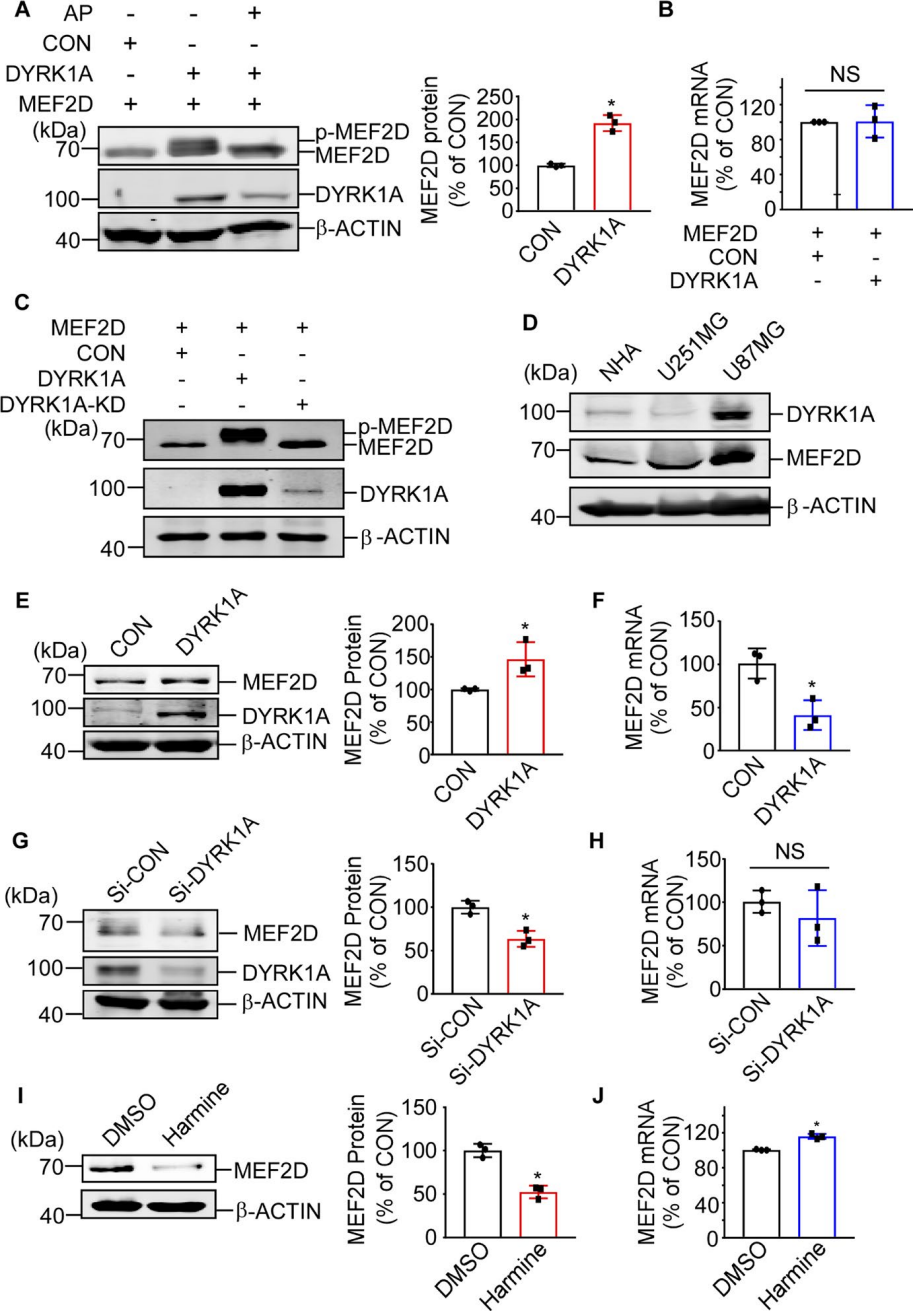
DYRK1A increases MEF2D protein level. (A) HEK293 cells were co‐transfected with MEF2D expression vector together with DYRK1A expression vector or the control vector. Alkaline phosphatase was applied into the cell lysate. Both MEF2D and DYRK1A were detected with anti‐flag antibody. (B) Transfection condition was the same as (A). RNA was isolated 48 h after transfection. Real‐time PCR was performed to examine MEF2D mRNA expression. (C) HEK293 cells were co‐transfected with MEF2D expression vector together with DYRK1A expression vector, DYRK1A kinase dead (DYRK1A‐KD) and the control vector, respectively. MEF2D and DYRK1A were detected by anti‐flag antibody. (D) MEF2D and DYRK1A protein expressions were examined among different glioblastoma cells. MEF2D and DYRK1A were detected with anti‐MEF2D and anti‐DYRK1A antibodies separately. (E) U87MG cells were transfected with DYRK1A expression vector and the control vector. MEF2D and DYRK1A were detected with anti‐MEF2D and anti‐DYRK1A antibodies. (F) Real‐time PCR was performed to examine the mRNA level of MEF2D in (E). (G) Si‐CON and Si‐DYRK1A were transfected into U87MG cells to silence DYRK1A. MEF2D and DYRK1A were detected with anti‐MEF2D and anti‐DYRK1A antibodies. (H) Real‐time PCR was performed to examine the mRNA level of MEF2D in (G). (I) Harmine, a specific inhibitor of DYRK1, was used to treat U87MG cells for 24 h with the concentration at 10 μmol/L. (J) Real‐time PCR was performed to examine the mRNA level of MEF2D in (I). Values represent means ± SD; n = 3; *P < .05 by non‐parametric *t* test

DYRK1A plays crucial roles in brain function and tumorigenesis. Biological activity of DYRK1A is generally ascribed to the phosphorylation of substrates. We find that overexpression of DYRK1A in HEK293 cells leads to a significant increase of MEF2D protein but not mRNA (Figure 1A,B). DYRK1A greatly elevates MEF2D protein level to 189 ± 2% compared with the control. Remarkably, molecular weight of MEF2D is partially changed by DYRK1A which could be reversed by incubation with alkaline phosphatase (Figure 1A). Mutation at Lys188 was confirmed to render DYRK1A catalytically inactive.^46^ DYRK1A‐K188R (DYRK1A‐KD) is used as negative control of wild type DYRK1A. As expected, no MEF2D bands with higher molecular weight are observed in the presence of DYRK1A‐KD (Figure 1C). The results indicate a possible phosphorylation of MEF2D by DYRK1A. According to previous research, MEF2D is abundant in many different tumour tissues.^8^, ^9^, ^10^, ^11^, ^12^ We then examine MEF2D expression in glioblastoma cell lines. The result shows both MEF2D and DYRK1A expressions are significantly higher in U87MG cells compared with U251MG cells and NHA cells (Figure 1D). In U87MG cells, MEF2D protein expression is induced by DYRK1A overexpression. The increase of MEF2D protein level reaches to 146.34 ± 20% compared with the control (Figure 1E). Silencing DYRK1A by siRNA leads to decreased MEF2D protein to 62.58 ± 6.6% compared with the control (Figure 1G). Harmine, a specific inhibitor of DYRK1, evidently decreases MEF2D protein level to 52 ± 5.6% compared with the control, which further verifies the induction of MEF2D protein by DYRK1A (Figure 1I). To exclude the increase of MEF2D by DYRK1A is via transcription regulation, mRNA of MEF2D was detected in cells with DYRK1A overexpression or down‐regulation. Quantitative real‐time PCR results reveal a reduction of mRNA level of MEF2D by DYRK1A overexpression in U87MG cells (Figure 1F) and no significant changes (Figure 1H) or slight increase (Figure 1J) are observed in cells with DYRK1A down‐regulated, indicating the elevation of MEF2D protein by DYRK1A might undergo a post‐translational modulation.

### 3.2 DYRK1A directly interacts with MEF2D

**FIGURE 2:**
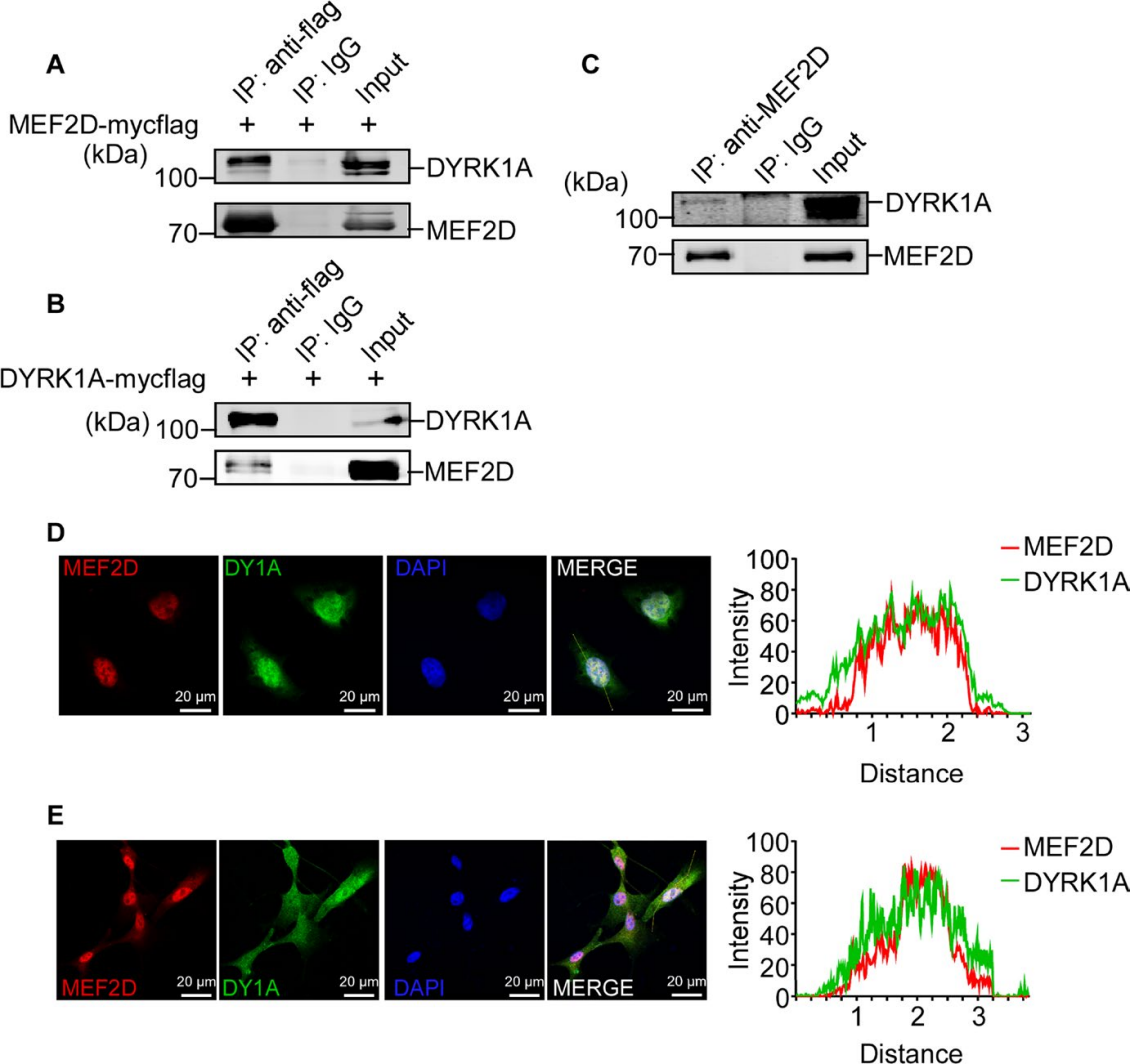
Interaction between DYRK1A and MEF2D. (A) HEK293 cells were transfected with MEF2D expression vector fused with myc and flag tags. Co‐immunoprecipitation (Co‐IP) with anti‐flag antibody was achieved to examine the specific binding of MEF2D and DYRK1A. DYRK1A was detected by anti‐DYRK1A antibody. (B) HEK293 cells were transfected with DYRK1A expression vector fused with myc and flag tags. Anti‐flag antibody was used for IP. MEF2D was detected by anti‐MEF2D antibody. (C) Cell lysates from U87MG cell lysate were immunoprecipitated with anti‐MEF2D antibody. DYRK1A was detected by anti‐DYRK1A antibody. (D) HEK293 cells were co‐transfected with MEF2D‐RFP and pEGFP‐DYRK1A vectors. Confocal microscopy was used to examine the co‐localization of exogenous DYRK1A and MEF2D in HEK293 cells. Co‐localization analysis was performed by ImageJ. (E) Confocal imaging was performed in U87MG cells. Anti‐MEF2D and anti‐DYRK1A antibodies were used to detect endogenous MEF2D and DYRK1A. Co‐localization analysis was performed by ImageJ

Since MEF2D expression is up‐regulated by DYRK1A, the interaction between them needs to be further addressed. pCMV6‐entry‐MEF2D is transfected into HEK293 cells. Co‐immunoprecipitation (Co‐IP) by anti‐flag antibody shows that MEF2D directly pulls down DYRK1A (Figure 2A). Co‐IP is also applied to HEK293 cells transfected with pCMV6‐entry‐DYRK1A as well. Result verifies a specific pull down of MEF2D by DYRK1A (Figure 2B). Interaction of endogenous MEF2D and DYRK1A is confirmed in U87MG cells by Co‐IP (Figure 2C). To further explore whether DYRK1A is co‐localized with MEF2D, immunofluorescence (IF) is performed in both HEK293 cells and U87MG cells. The images acquired on confocal microscope exhibit a strong co‐localization of exogenous DYRK1A and MEF2D in HEK293 cells mainly in the nucleus (Figure 2D). Similar as it's in HEK293 cells, endogenous MEF2D and DYRK1A are co‐localized in U87MG cells as well (Figure 2E). Results above provide strong evidence that DYRK1A and MEF2D might physically interact with each other.

### 3.3 DYRK1A is responsible for phosphorylation of MEF2D at Ser251

**FIGURE 3:**
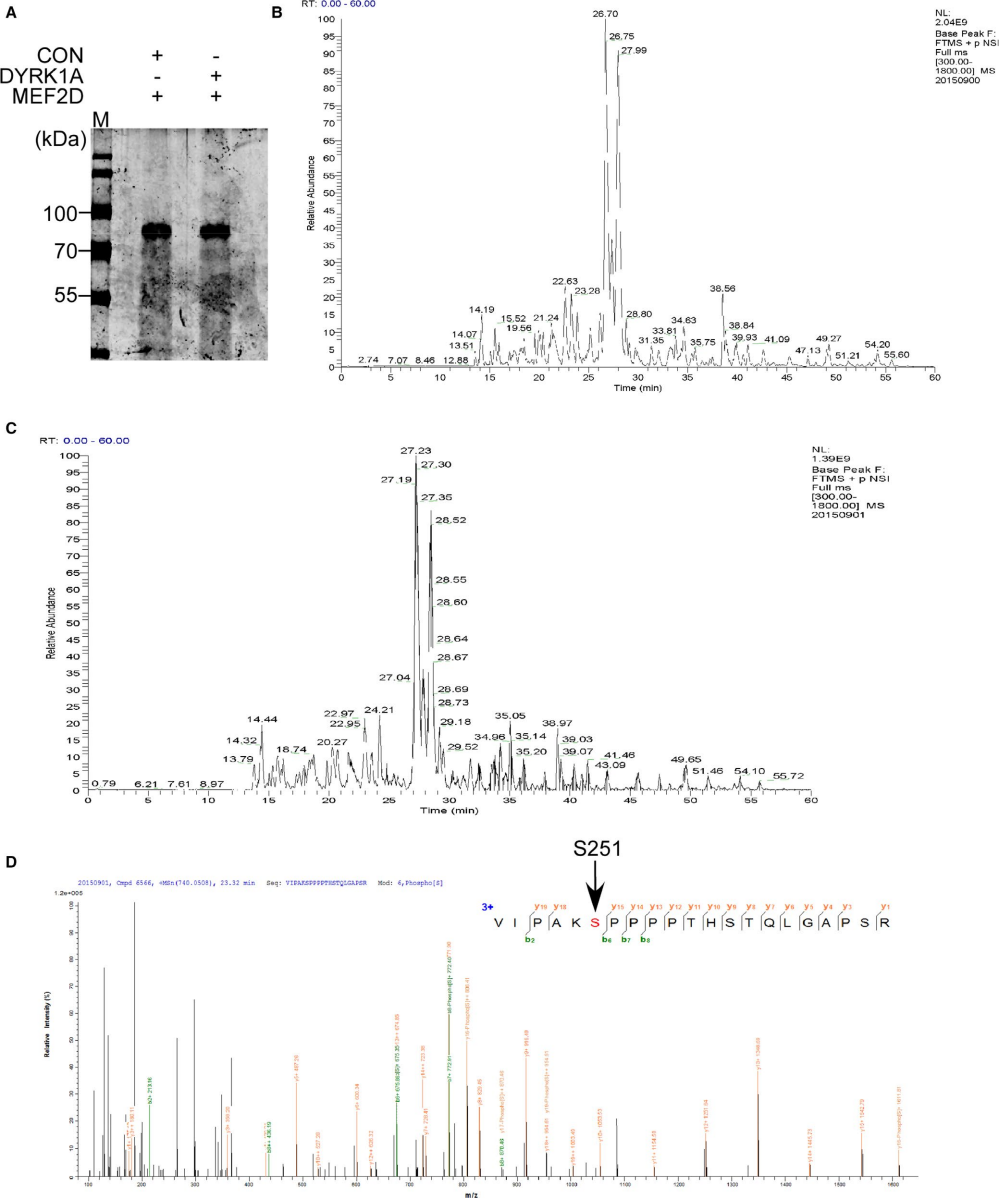
Mass spectrometric analysis of phosphorylation of MEF2D by DYRK1A in vitro. (A) Purified recombinant human MEF2D and DYRK1A proteins were used for in vitro kinase assay. SDS‐PAGE was performed to obtain MEF2D protein after in vitro kinase assay. (B) Basepeak result of MS for MEF2D without DYRK1A. (C) Basepeak result of MS for MEF2D with DYRK1A. (D) Mass spectrometric analysis of MEF2D phosphorylation. Matching data of 5 fragments indicate the same phosphorylation modification at Serine 251, which was highlighted with arrow

Considering possible phosphorylation of MEF2D by DYRK1A, phosphorylation sites on MEF2D need to be identified. Recombinant protein purification combined with in vitro kinase assay was applied to obtain phosphorylated MEF2D. The band was clearly detected by Coomassie Brilliant Blue staining on SDS‐PAGE (Figure 3A). The gel was fixed and the gel location of MEF2D band was cut for mass spectrometry (MS) analysis. Analysis by Mascot showed that MEF2D was phosphorylated at Ser251 upon DYRK1A based on m/z of five different peptides after in‐gel digestion. The phosphorylation was not detected from control sample (Figure 3B‐D).

### 3.4 Phosphorylation by DYRK1A inhibits transcriptional activity of MEF2D

**FIGURE 4:**
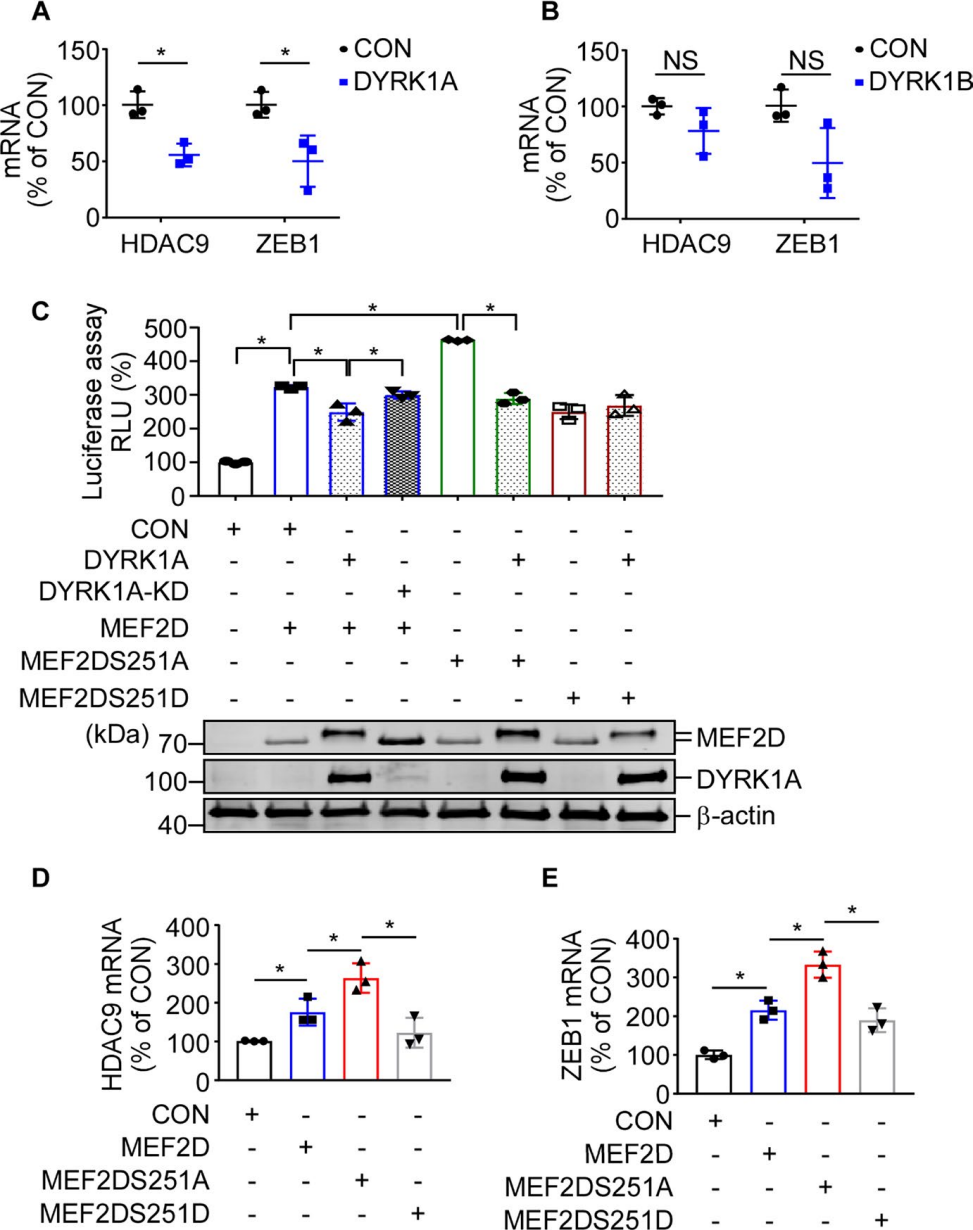
Inhibition of transcriptional activity of MEF2D by DYRK1A. (A) U87MG cells were transfected with DYRK1A expression vector or the control vector. RNA was isolated 48 h after transfection. Real‐time PCR was performed to examine mRNA expression of HDAC9 and ZEB1. (B) U87MG cells were transfected with DYRK1B expression vector or the control vector. mRNA levels of HDAC9 and ZEB1 were detected by real‐time PCR. (C) Luciferase assay was performed to examine the transcriptional activity of MEF2D. (D) HDAC9 mRNA level was detected in U87MG cells upon MEF2D variants. (E) ZEB1 mRNA level was detected in U87MG cells. Values represent means ± SD; n = 3; *P < .05 by non‐parametric *t* test or two‐way ANOVA analysis

Accumulative studies have demonstrated that phosphorylation of MEF2D could affect its transcriptional activity.^18^, ^24^, ^47^ HDAC9 and ZEB1 were directly regulated by MEF2D and could be used to exemplify transcriptional activity of MEF2D.^48^, ^49^ Real‐time PCR is performed to examine the mRNA expression of HDAC9 and ZEB1 with or without DYRK1A overexpression. The result reveals significant decreases of HDAC9 and ZEB1 mRNA level induced by DYRK1A (Figure 4A). DYRK1B, another member of DYRK family, is a paralog of DYRK1A that shares 85% sequence identity in the catalytic domain and the neighbouring DH box. The effect of DYRK1B on co‐regulating MEF2D transcriptional activity was investigated by examining HDAC9 and ZEB1 mRNA expression. The result shows a mild decrease of HDAC9 and ZEB1 mRNA level upon DYRK1B overexpression, which manifests DYRK1B may not be a direct co‐regulator to MEF2D transcriptional activity (Figure 4B).

Dual‐luciferase assay is performed in HEK293 cells transfected with MEF2D responsive element vector (3xMREluc), which includes conserved sequences recognized by MEF2. MEF2D significantly induces luciferase activity of MREluc, which is reversely attenuated by DYRK1A but restored by DYRK1A‐KD in which the K188R mutation abolishes the kinase activity of DYRK1A (Figure 4C). In order to better understand how MEF2D transcriptional activity is affected by DYRK1A phosphorylation, Ser251A (Ser‐Arg) and Ser251D (Ser‐Asp) mutation vectors are constructed and included in luciferase assay for comparison. As expected, the result shows a higher transcriptional activity of MEF2D‐S251A compared with wild type MEF2D (lane 5 compared to lane 2, Figure 4C) but a relatively lower transcriptional activity of MEF2D‐S251D (lane 7 compared to lane 2 of Figure 4C). This result is also confirmed by qPCR in U87MG cells. MEF2D‐S251A markedly increases HDAC9 and ZEB1 mRNA expression compared with wild type MEF2D while MEF2D‐S251D modestly abolishes the regulation enhancement (Figure 4D,E). These results suggest that transcriptional activity of MEF2D is significantly inhibited by DYRK1A through phosphorylation at Ser251.

### 3.5 Phosphorylation by DYRK1A facilitates its nuclear export without affecting protein stability

**FIGURE 5:**
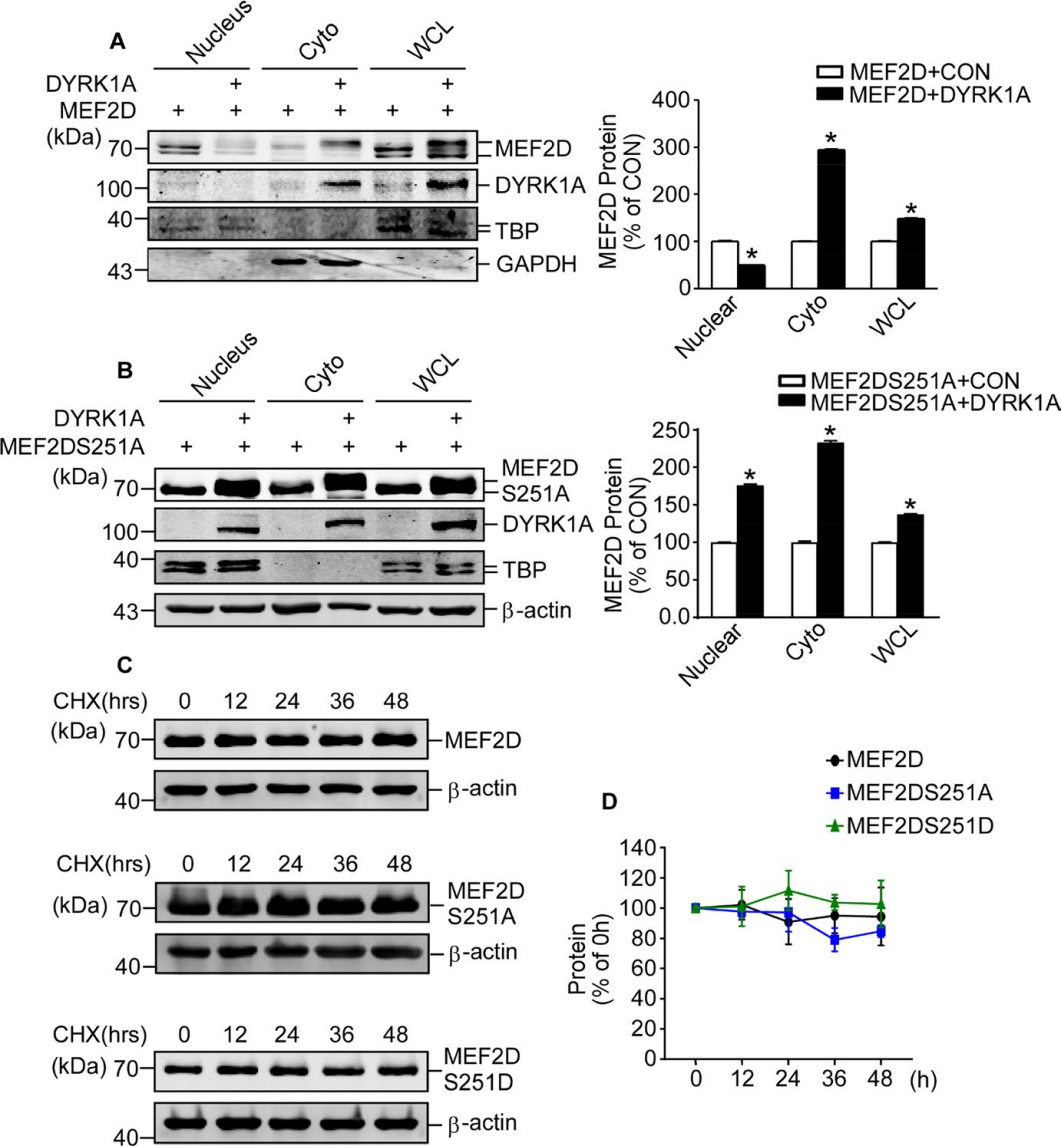
DYRK1A alters subcellular localization of MEF2D without affecting protein stability. (A) Subcellular fractionation was performed on HEK293 cells that were co‐transfected with MEF2D expression vector together with DYRK1A or the control vector. Anti‐MEF2D and anti‐DYRK1A antibodies were applied. (B) Subcellular fractionation was performed on HEK293 cells that were co‐transfected with MEF2DS251A vector with DYRK1A or the control vector. Anti‐MEF2D and anti‐DYRK1A antibodies were applied. (C) CHX pulse‐chase assay was performed to HEK293 cells that were transfected with MEF2D, MEF2DS251A and MEF2DS251D vectors. Final concentration of CHX is 150 μg/mL. (D) Comparison of wild type MEF2D to MEF2DS251A and MEF2DS251D in CHX pulse‐chase assay. Values represent means ± SD; n = 3; *P < .05 by two‐way ANOVA analysis

DYRK1A increases MEF2D expressions by phosphorylation at Ser251, but decreases the transcriptional activity of MEF2D. To gain further insights into the regulation of MEF2D by DYRK1A, subcellular localization of MEF2D by DYRK1A is investigated. Subcellular fractionation is performed in HEK293 cells with MEF2D and DYRK1A overexpression to assess the intracellular transport of MEF2D. As can be observed, MEF2D protein level is tremendously decreased in the nucleus but increased in the cytoplasm and whole cells (Figure 5A). Reduced MEF2D protein in the nucleus might partially explain the reduction of MEF2D transcriptional activity by DYRK1A. Subcellular fractionation with MEF2D‐S251A reveals a restored MEF2D protein level in nuclear fraction, further confirmed that DYRK1A phosphorylation of MEF2D could facilitate the MEF2D export from the nucleus to the cytoplasm (Figure 5B). These results support that DYRK1A inhibits import of MEF2D into the nucleus. To better understand whether phosphorylated MEF2D undergoes faster degradation, cycloheximide (CHX) pulse‐chase assay is performed in cells transfected with MEF2D, MEF2DS251A and MEF2DS251D. Unexpectedly, both MEF2DS251A and MEF2DS251D exhibit similar degradation rate as wild type MEF2D (Figure 5C,D). It suggests that phosphorylation at Ser251 may not affect protein stability.

## 4 DISCUSSION

MEF2D is highly expressed in various malignancies and participating in biological functions of cancer cells. Aberrant MEF2D expression and phosphorylation affect cancer cell normal function. In this study, we report that MEF2D is phosphorylated by DYRK1A in both HEK293 cells and U87MG cells. Phosphorylation by DYRK1A might contribute to MEF2D protein accumulation. Ser251 is identified by MS analysis as the phosphorylation site by DYRK1A. Transcriptional activity of MEF2D is significantly inhibited by DYRK1A. These results are further confirmed by luciferase assay with mutated MEF2DS251A, MEF2DS251D and DYRK1A‐KD. Phosphorylation at Ser251 affects MEF2D nuclear export, indicating that MEF2D phosphorylation has impact on nuclear transport.

Our previous work has demonstrated that MEF2D up‐regulates DYRK1A expression through activation of DYRK1A gene transcription.^14^ Interestingly, we happened to notice that mRNA expression of exogenous MEF2D was not changed by DYRK1A, which may be ascribed to overwhelming copies of transfected MEF2D. Conversely, MEF2D mRNA was down‐regulated by DYRK1A in U87MG cells. These results suggest that elevated MEF2D protein expression by DYRK1A might be independent of mRNA expression. Other underlying mechanism responsible for alteration of MEF2D mRNA expression by DYRK1A needs to be further investigated. Remarkably, co‐localization of MEF2D and DYRK1A is observed in most cells but not in a small number of cells. This renders possibility of their direct interaction at certain interphases during cell cycle.

DYRK1B is the most closely related homologue of DYRK1A. Silencing DYRK1A results an increase in DYRK1B.^50^ DYRK1A and DYRK1B may phosphorylate the same substrates and function correlatively.^51^, ^52^ We simply evaluate modulation to MEF2D by DYRK1B by detecting mRNA expression of MEF2D target genes. Our results show that DYRK1B mildly decreases mRNA expression of HDAC9 and ZEB1. DYRK1B might regulate MEF2D in a similar way as DYRK1A but less potent. Relevant mechanism needs further exploration.

Phosphorylation of MEF2A and MEF2D at certain amino acids elicits attenuated DNA binding ability, reduced transcriptional activity and caspase‐dependent cleavage to fragments containing N‐terminal DNA binding domains and C‐terminal transactivation domains.^20^ In our study, we detect higher luciferase activity of MEF2DS251A and lower luciferase activity of MEF2DS251D compared with wild type MEF2D in dual‐luciferase assay. It proves Ser251 is vital to MEF2D transcriptional activity. The activity of MEF2D was regulated by chaperone‐mediated autophagy.^25^ Phosphorylation by Cdk5 is sufficient to promote degradation of MEF2D through activation of caspase‐3.^22^ No significant change to the caspase‐dependent cleavage of MEF2D by DYRK1A was detected (data not shown). Cytoplasmic MEF2D was detected in subcellular fractionation assay in the presence of DYRK1A. MEF2D is present in rodent neuronal mitochondria and enhances complex I activity by promoting ND6 transcription.^53^ Levels of cytoplasmic MEF2D, significantly lower in the nigral neurons with alpha‐synuclein inclusions, might also be involved in PD pathogenesis.^27^ Hence, further scientific investigation might be necessary to better understand the function of phosphorylated MEF2D in the cytoplasm. By cycloheximide pulse‐chase assay, we notice all MEF2D variants are extremely stable after being treated with cycloheximide for 48 hours. This might rely on the pro‐survival functions of MEF2D against cell death.^20^, ^54^, ^55^

Mass spectrometry screening only identifies Ser251 in MEF2D as phosphorylation site by DYRK1A. Our results show that MEF2D‐S251A protein expression is still increased by DYRK1A overexpression compared with the control, suggesting Ser251 is not the unique phosphorylation site induced by DYRK1A. But the results of our study validated that Ser251 was a crucial functional site of MEF2D in glioma cells. Phosphorylation of MEF2D at diverse sites determined its transactivation, indicating that more phosphorylation sites needed to be identified to enrich relevant mechanisms. Aberrant expression of DYRK1A is detected in glioblastomas tissue. Inhibition of DYRK1A promotes EGFR degradation in primary glioblastoma cell lines and neural progenitor cells, sharply reducing the self‐renewal capacity of normal and tumorigenic cells.^29^ Another study found that DYRK1A and DYRK1B kinases phosphorylate ID2 on threonine 27 (Thr27), leading to HIF2α destabilization, loss of glioma stemness and inhibition of tumour growth.^30^ As DYRK1A is a potential therapeutic target to glioblastoma growth, more work needs to be achieved to profoundly elucidate underlying mechanism.

## CONFLICT OF INTEREST

The authors declare that they have no conflicts of interest with the contents of this article.

## AUTHOR CONTRIBUTIONS

**Pin Wang:** Investigation (lead); methodology (equal); writing‐original draft (lead). **Xiulian Sun:** Methodology (lead); project administration (lead); writing‐review & editing (lead). **Juan Zhao:** Investigation (equal).

## Data Availability

The data that support the findings of this study are openly available in this article.
